# A Hydrogen Bonds-Crosslinked Hydrogels With Self-Healing and Adhesive Properties for Hemostatic

**DOI:** 10.3389/fbioe.2022.855013

**Published:** 2022-04-14

**Authors:** Han Yu, Qiaohong Xiao, Guilin Qi, Feixiang Chen, Biyue Tu, Suo Zhang, Yinping Li, Yun Chen, Hui Yu, Peng Duan

**Affiliations:** ^1^ Department of Pathology, Xiangyang No.1 People’s Hospital, Hubei University of Medicine, Xiangyang, China; ^2^ Key Laboratory of Zebrafish Modeling and Drug Screening for Human Diseases of Xiangyang City, Department of Obstetrics and Gynaecology, Xiangyang No.1 People’s Hospital, Hubei University of Medicine, Xiangyang, China; ^3^ Department of Pathophysiology and Hubei Province Key Laboratory of Allergy and Immunology, School of Basic Medical Sciences, Wuhan University, Wuhan, China; ^4^ Department of Biomedical Engineering and Hubei Province Key Laboratory of Allergy and Immunology, School of Basic Medical Sciences, Wuhan University, Wuhan, China; ^5^ Fourth Clinical College, Hubei University of Medicine, Shiyan, China

**Keywords:** hydrogel, hydrogen bond, adhesive, self-healing, hemostatic

## Abstract

Hydrogels with adhesive properties have the potential for rapid haemostasis and wound healing in uncontrolled non-pressurized surface bleeding. Herein, a typical hydrogen bond-crosslinked hydrogel with the above functions was constructed by directly mixing solutions of humic acid (HA) and polyvinylpyrrolidone (PVP), in which the HA worked as a crosslinking agent to form hydrogen bonds with the PVP. By altering the concentration of HA, a cluster of stable and uniform hydrogels were prepared within 10 s. The dynamic and reversible nature of the hydrogen bonds gave the HA/PVP complex (HPC) hydrogels injectability and good flexibility, as well as a self-healing ability. Moreover, the numerous functional groups in the hydrogels enhanced the cohesion strength and interaction on the interface between the hydrogel and the substrate, endowing them with good adhesion properties. The unique chemical composition and cross-linking mechanism gave the HPC hydrogel good biocompatibility. Taking advantage of all these features, the HPC hydrogels obtained in this work were broadly applied as haemostatic agents and showed a good therapeutic effect. This work might lead to an improvement in the development of multifunctional non-covalent hydrogels for application to biomaterials.

## Introduction

Hydrogels, which are generated by exploiting covalent and/or noncovalent interactions between polymeric components, have been extensively studied due to their unique “human tissue-like” properties, such as their toughness, permeability, biocompatibility, self-healing ability and wettability (Li D. et al., 2020; Poupart et al., 2020; Chen et al., 2021; Kim et al., 2021). In particular, hydrogels with self-healing ability can spontaneously repair the cracked network when deformation occurs, without external stimuli, and maintain their efficacy and integrity in complex physiological environments; consequently, they have attracted great attention in recent years. Self-healing hydrogels have demonstrated tremendous superiority in a range of fields, including drug delivery, tissue engineering and cell therapy. Many strategies have been explored to develop self-healing hydrogels (Liu and Hsu 2018; Lin et al., 2021; Kim et al., 2022). The introduction of dynamic and reversible noncovalent interactions, including coordination bonds, ionic bonds, host-guest interaction and hydrogen bonds, could endow a hydrogel with a self-healing ability, as well as stimuli-responsive and adaptive properties (Cao et al., 2018; Huang et al., 2018; Yan et al., 2020). Among the wide ranges of noncovalent bonds, hydrogen bonds are commonly found in natural systems, due to their bonding strength and low toxicity, and have been widely used to design functional hydrogels (Chen et al., 2016; Zhang et al., 2022). Moreover, the hydrogen bonds in a hydrogel serve as sacrificial bonds and show an excellent toughening effect, by effectively dissipating external energy (Zhang et al., 2018). Hydrogen bond-crosslinked hydrogels have attracted particular interest for polymeric biomaterials.

However, hydrogen bonds existing in a hydrogel might easily be disturbed by water molecules, which may severely reduce their stability and crosslinking effect in an aqueous environment (Long et al., 2018). Thus, the preparation of functional hydrogels based on hydrogen bonds is still challenging. Various strategies have been explored to develop hydrogen bond crosslinking systems that have high efficiency. In recent years, researchers have found that the introduction of hydrophobic domains can effectively improve the stability of the hydrogen bond (Zhang X. et al., 2019; Dompe et al., 2019; Wang et al., 2019; Criado-Gonzalez et al., 2020) For instance, Wu and co-workers have developed hydrogels with robust hydrogen bond networks using methacrylamide (MAAm) and methacrylic acid (MAAc) as raw monomers. The carboxyl and amide groups in the hydrogel formed stable hydrogen bond interactions that were effectively promoted by the hydrophobic methyl groups. The obtained hydrogels possessed excellent mechanical properties (elastic modulus: 2.3–217.3 MPa; fracture strength: 1.2–8.3 MPa; fracture strain: 200–620%), good shape memory performance, and cycling stability (Wang et al., 2019). According to this principle, molecules with both hydrophobic groups and active functional groups, including tannic acids (TA), lignin, and silicotungstic acid, were widely adopted as cross-linking agents to prepare hydrogen bond-crosslinked hydrogels (Zhang X. et al., 2019; Zhang Z.-X. et al., 2019; Peng et al., 2020). Humic acid (HA) is a degradation product of animals and plants, and contains almost 50% of the natural organic carbon source on earth (Ratie et al., 2021; Venezia et al., 2021). It is a versatile material in agricultural, environmental and healthcare applications due to its rich reserves, low cost and degradable features (Hou et al., 2018). In addition, the coexistence of hydrophobic groups and active functional groups make HA a potential candidate as a hydrogen bond crosslinking agent.

Hydrogels with a high adhesion strength to various substrates have also aroused growing interest in many practical applications, such as wound dressing, haemostatic agents and soft electronic devices (He et al., 2018; Wei et al., 2019; Zhang et al., 2020). Previous works have reported diverse strategies for the preparation of multifunction adhesive hydrogels, including chemical anchorage, physical adhesion, mechanical interlocking and bio-inspired means (Karami et al., 2018; Yang et al., 2018). Physical adhesion interactions, like hydrogen bonds, electrostatic and hydrophobic interactions, at the interface between the hydrogel and substrate were studied due to their multipurpose and reusable abilities (Xing et al., 2021; Li et al., 2022). For example, Guo developed adhesive hydrogels based on derivatives of hyaluronic acid, poly (ethylene glycol)-*co*-poly (glycerol sebacate) and cuttlefish melanin nanoparticles. The amino and catechol groups formed multiple interactions with tissue, which enabled the attachment to the skin surface to be maintained (Li et al., 2022).

Considering the requirements of hydrogel-based biomaterials, a novel HA/polyvinylpyrrolidone (PVP) complex (HPC) hydrogel with self-healing and adhesion properties has been developed in this work. The gelation process is forthright and rapid, using one-step mixing of aqueous solutions of HA and PVP. The construction of the HPC hydrogel is driven by the hydrogen bonds between HA and PVP, which are readily formed and adjusted. The dynamic and reversible hydrogen bond distributed in the hydrogel promotes the formation of a dynamic crosslinking network and endows the HPC hydrogel with good mechanical properties, injectability, self-healing properties and high adaptation to irregular substrates. In addition, the HPC hydrogels demonstrate good adhesion strength to multiple materials, which originates from the hydrogen bond between the HPC hydrogel and the surface of the substrates. The biologically compatible composition and gelation mechanism endows the HPC hydrogels with good biocompatibility, and they show good efficacy when serving as haemostatic agents in the bleeding model. The results suggest that this kind of self-healing, adhesive, injectable and haemostatic hydrogel exhibits great potential for application to biomedical materials.

## Materials and Methods

### Materials

PVPs with Mw 8,000, 24,000, and 1,300,000 Da were bought from Aladdin (Shanghai, China). HA, NaOH, HCl, urea and dimethyl sulfoxide (DMSO) were bought from Sinopharm (Shanghai, China) and were used without any purification. Porcine skin was provided by the supermarket and was cleaned before use.

### Preparation of HA/PVP complex Hydrogel

HA and PVP were separately dissolved in water. The two solutions were then uniformly mixed and vigorously shaken. The pH value was adjusted to 7 using 0.1 M HCl aqueous solution. The solutions tended to gelation, and the HPC hydrogel was finally formed. The concentration of PVP was 15 wt%.

### Characterizations

All tensile tests of the HPC hydrogel were performed on a universal tensile tester (Instron machine 3343) at room temperature. The samples were stretched at a deformation rate of 100 mm/min. The toughness of the HPC was calculated by integrating the area under the stress-strain curve. The elastic modulus was determined by the slope of the initial stress-strain curves.

### Dynamic Rheological Measurements of HA/PVP Complex Hydrogels

Dynamic rheological measurements were taken on the ARES RFSIII rheometer (TA Instruments, United States) using flat plates (40 mm). The gap spacing was set to 2 mm. Oscillatory frequency sweep measurements were carried out to determine the storage modulus (G′) and loss modulus (G″) of the hydrogels at 25°C. The strain was set to 10%, with the shear frequency (*ω*) ranging from 0.1 to 100 rad/s. The temperature sweep test was conducted under the fixed frequency and strain of 10 rad/s and 10%, respectively.

### Adhesion Properties of HA/PVP Complex Hydrogels

Adhesion properties were measured using the lap-shear tension loading test. The HPC hydrogels were sandwiched between the overlap areas of two pieces of the substrate (for example, porcine skin with a regular rectangular shape), and the contact areas were measured and calculated. The samples to be tested were placed at room temperature for 15 min prior to the tensile test, which was carried out using a stretching rate of 20 mm/min. The adhesion strength of the samples to other materials was tested in the same way.

### Cytotoxicity of HA/PVP Complex Hydrogels

The biocompatibility of the HPC hydrogels was tested with the Calcein-AM/PI double staining method. The HPC hydrogels were freeze-dried and sterilized before testing. The HPC hydrogels were seeded with L929 cells with a density of 4 × 10^4^ cells per sample, incubated for 24 h, and the cells then stained with calcein-AM and PI, respectively. The phalloidin staining evaluation was also performed according to the kit manufacturer’s protocols. The cell morphology was observed by a laser confocal microscope (FV3000, Olympus).

### Haemolysis Evaluation of HA/PVP Complex Hydrogels

The haemolysis evaluation was conducted to assess the blood biocompatibility of the HPC hydrogel. In brief, rabbit blood was collected and diluted with PBS solution (2.5 ml). The series of HPC hydrogels were incubated in PBS solution (8 ml) in a water bath (37°C) for 1 h. Then, 0.2 ml diluted fresh blood was slowly added into the mixed solutions. The obtained mixture was incubated for 60 min, transferred into a centrifuge tube and centrifuged at 5,000 rpm for 8 min. Optical photos of the haemolysis results were taken. At the same time, the absorbance of the supernatant was measured at 540 nm using a UV spectrometer (UV-9200/VIS-7220G, China). A natural polymer-based hydrogel were chosen to be the control group. The haemolysis ratio was calculated as follows:

$$\text{Haemolysis ratio \% }=\left[\left({\text{A}}_{\text{S}}-{\text{A}}_{\text{N}}\right)/\left({\text{A}}_{\text{P}}-{\text{A}}_{\text{N}}\right)\right]\text{×}100\text{\%}$$

where A_S_, A_P_, and A_N_ are the optical density values from the hydrogel, PBS solution, and deionized water, respectively.

### Biohistocompatibility Evaluation of HA/PVP Complex Hydrogel

The HPC-2 hydrogel was transplanted into rats, and the heart, liver, spleen, lung and kidney tissues of the rats were harvested 14 days later. These tissue samples were observed in the HE staining.

### Haemostatic Performance and Wound Healing Behaviour of HA/PVP Complex Hydrogels

The liver bleeding model of the rat was selected to evaluate the *in vivo* haemostatic ability of HPC hydrogels. Before the animal experiments, male SD rats were subjected to abdominal anaesthesia. The anaesthetised rats were immobilized, and the abdomen of the rats was cut open with a scalpel to expose the liver. The liver of the rat was then cut open with a scalpel and a filter paper placed under the liver to collect the blood from the rat. HPC-2 hydrogel, with a weight of 2 g, was appended to the liver to treat the injury. No treatment was given in the control group.

The haemostatic index was tested through the whole blood coagulation experiment. Fresh samples of the rats’ arterial blood were mixed evenly with gelatin sponge and freeze-dried hydrogel samples. CaCl_2_ solution (20 μL, 0.2 mol/L) was then added and the mixture placed in a water bath (37°C). The light absorbance of the solutions at 540 nm was then measured, wherein deionized water was used as a control. The haemostatic index was calculated as follows:

$$\text{Haemostatic index}\left(\text{\%}\right)={\text{A}}_{\text{h}}/{\text{A}}_{\text{c}}\text{×}100\text{\%}$$

where A_h_ is the absorbency of HPC hydrogel group and A_c_ represents the absorbency of the control group.

### Statistical Analysis

All statistical analyses were performed with SPSS software (SPSS software version 12.0, SPSS, Chicago, IL, United States). The data were expressed as the mean ± standard deviation (SD). All experiments were repeated at least three times. Statistical analysis across multiple groups was performed using one-way analysis of variance (ANOVA), followed by Fisher’s LSD post-hoc test for homogeneity of variance. Two-sided *p* values of <0.05, <0.01 and <0.001 were considered as statistically significant or highly significant, respectively. Line graphs and bar graphs were generated using GraphPad PRISM Version 8.0 (San Diego, CA, United States).

## Results and Discussion

### Preparation of Various HA/PVP Complex Hydrogels

Various HPC hydrogels were successfully prepared by changing the HA content of the hydrogels. The specific parameters of the experiment are shown in Table 1.

**TABLE 1:** Composition and mechanical parameters of the prepared HPC hydrogels.

Samples	*c*_HA_ (wt%)	*c*_PVP_ (wt%)	Fracture Stress (KPa)	Fracture Strain (%)
HPC-1	6	15	11.8	776
HPC-2	8	15	18.9	643
HPC-3	10	15	17.0	403
HPC-4	4	15	-^c^	-^c^
HPC-5	6	12	-^c^	-^c^

*C*_HA_: Concentration of HA.

*C*_PVP_: Concentration of PVP.

c: Too weak to be meaningful.

**FIGURE 1:**
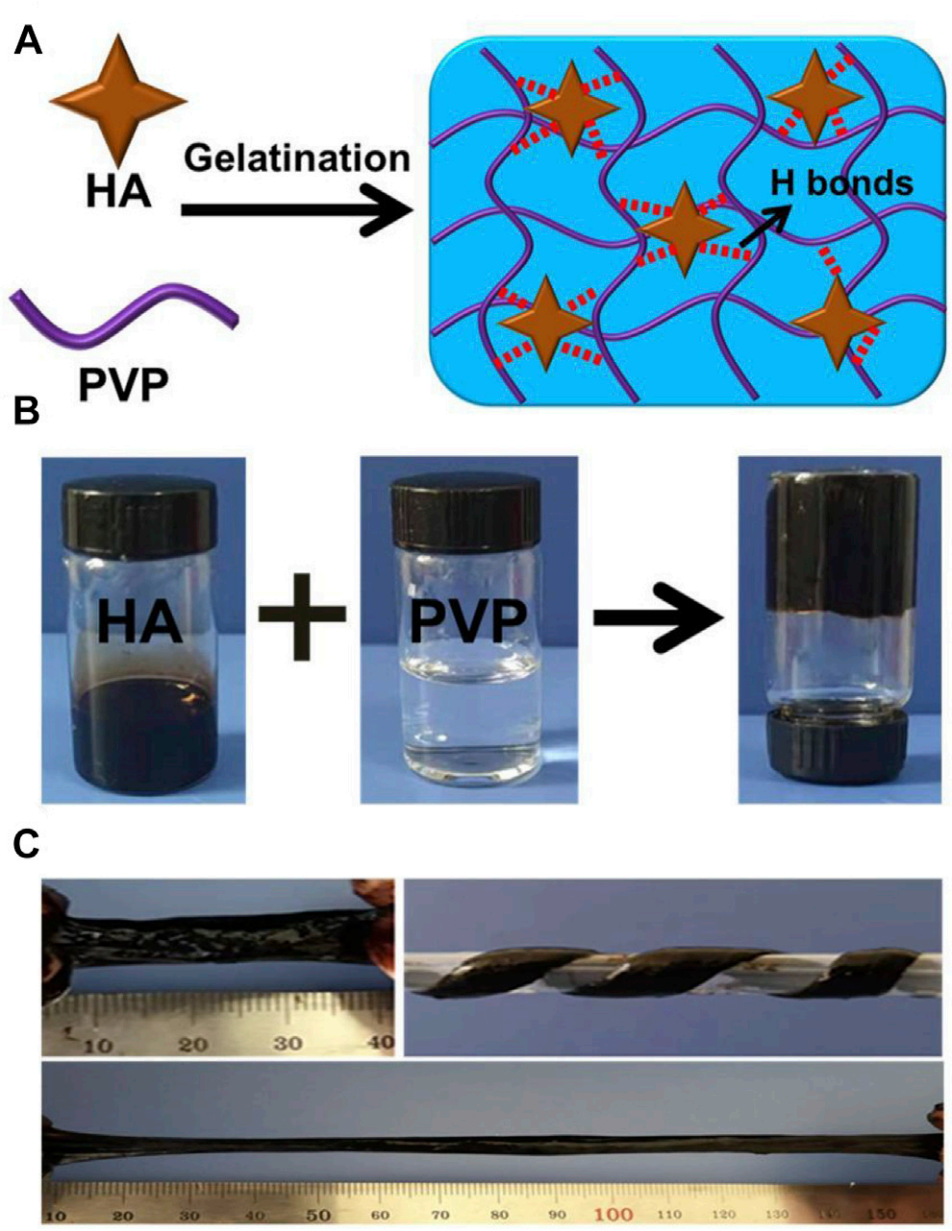
**(A)** Schematic illustration of HPC hydrogels crosslinked by HA in an ultrafast process. **(B)** Images showing the preparation process of the HPC hydrogels. **(C)** Photographs of the HPC hydrogel. The obtained HPC-2 hydrogel was uniform and could be easily stretched and twisted.

We observed a rapid gelation phenomenon based on the crosslinking effect of the hydrogen bonds between HA and PVP. Figures 1A,B show the fast and convenient manufacturing procedure of the HPC hydrogels. Different concentrations of HA and PVP solutions were prepared in advance. It is noteworthy that HA was dissolved in a weakly alkaline aqueous solution (pH = 9) to convert the carboxyl groups to carboxylate and improve its solubility in water. By simply mixing the prepared HA and PVP solutions together and adjusting the pH value of the mixture to neutral, viscous and flexible hydrogels with a black homogeneous appearance were finally obtained within 10 seconds (Figure 1C). Thus, the Mw of PVP we used in this We then adjusted the ratio of HA to PVP, to prepare various HPC hydrogels with different characteristics. One of the most important factors that influenced the state of the hydrogel was the molecular weight of the polymer. The molecular weights (Mw) of PVP played an important role in the formation of the hydrogel. When the Mw of PVP was low, the hydrogel could not form and only solutions were obtained (Supplementary Figure S1). The Mw of PVP chosen to prepare the HPC hydrogel was 1,300,000 Da. It is worth noting that the concentration of PVP and HA could also affect the gelation process, as adequate polymer chains and crosslinking density were required to form the hydrogel (Dompé et al., 2019). Only when the concentration of both PVP and HA exceeded a certain value could HPC hydrogels with satisfactory performance be obtained. For example, when the concentration of PVP was lower than 15 wt% or the concentration of HA was 4 wt%, the obtained HPC samples were too weak to be tested. Based on this principle, the HPC-2 hydrogel was successfully prepared using 8 wt% HA and 15 wt% PVP, which has the highest fracture stress (18.9 kPa) and appropriate fracture strain (643%). Thus, HPC-2 was chosen as the model material to further explore the gelation mechanism of the hydrogel (Table 1). The FT-IR of PVP and HPC-2 samples was given in Supplementary Figure S2. The chemical shift of amide groups on PVP (1,662 cm^−1^) decreased to 1,645 cm^−1^, indicating the existence of hydrogen bonding interaction between HA and PVP in HPC-2. When temperature increased, part of hydrogen bonds broke and the signal at around 1,644 cm^−1^ became weak. When the temperature returned, the signal recovered to original one, showing the “dynamics and reversibility” properties of hydrogen bonds. When DMSO, DMF, or urea was added, the hydrogen bonds broke and the HPC hydrogels changed to solutions. The HPC hydrogel also showed reversible responsivity to H^+^ and OH^−^, indicating the dynamic and reversible properties of the hydrogen bonds (Supplementary Figure S3). The swelling ratios of the HPC hydrogels were tested. The maximum swelling ratio gradually increased with the relative content of HA increased. Like other non-covalent supramolecular hydrogels, the HPC hydrogel would gradually disintegrate due to the hydration when immersed in water (Supplementary Figure S4).

### Rheological Properties of HA/PVP Complex Hydrogels

**FIGURE 2:**
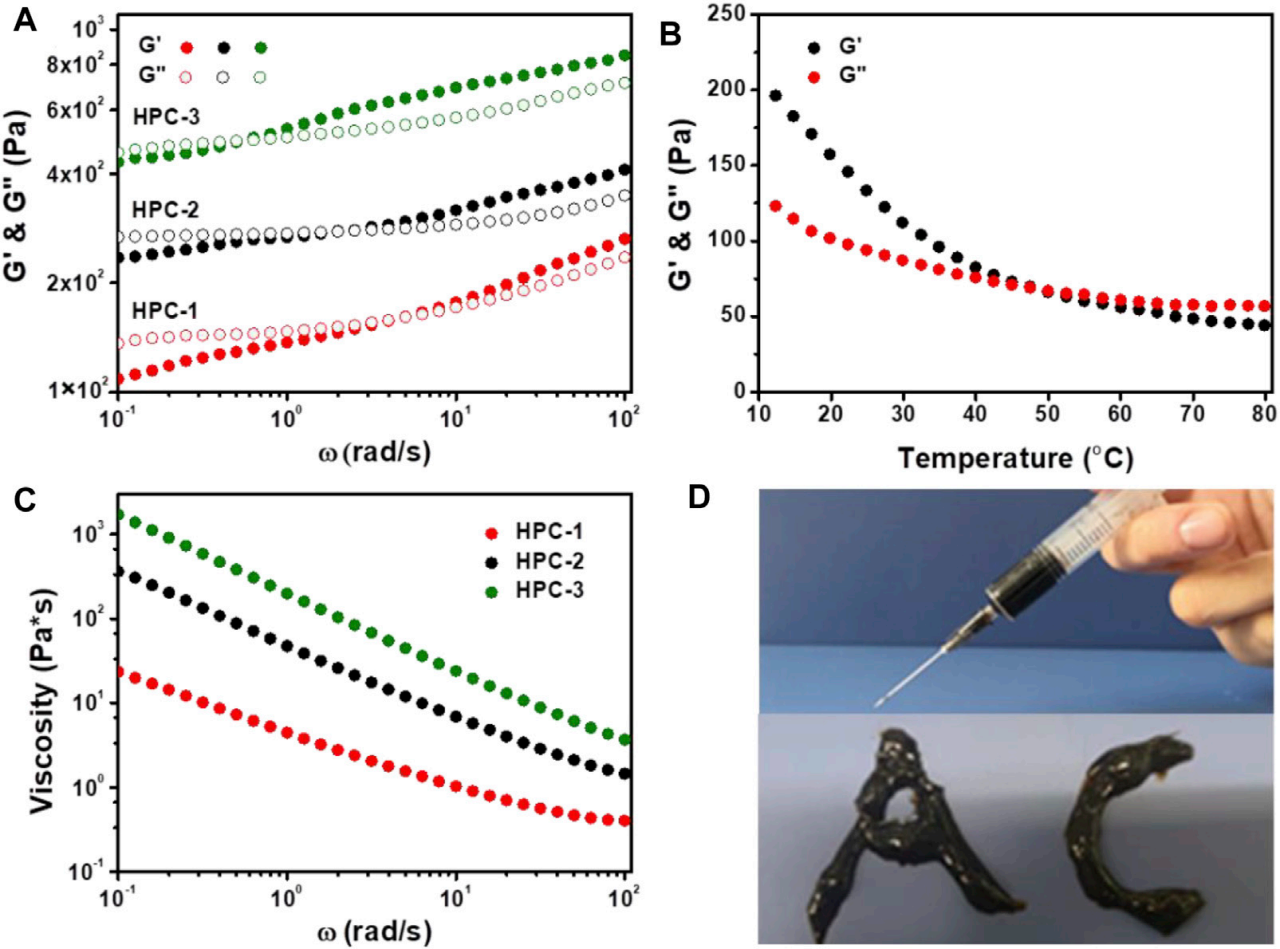
**(A)** G′ and G″ as a function of *ω* for the HPC hydrogels. **(B)** Temperature dependence of G′ and G″ for the HPC-2 hydrogel **(C)** The shear viscosity of HPC hydrogels as a function of ω. **(D)** HPC-2 hydrogel used as ink to write words.

Firstly, the strength and structural stability of the HPC hydrogels were evaluated using dynamic frequency sweep tests. As shown in Figure 2A, the phase change could be obviously observed in all three samples, which showed similar characteristics to other noncovalent hydrogels. When the *ω* values were low, G′ was lower than G″, and the HPC samples behaved like viscous fluids, due to the stress dissipation (Grindy et al., 2015). As the strain frequency increased, G′ gradually exceed G″, and the HPC hydrogels exhibited obvious elastic behaviour (Chaudhuri et al., 2016). In addition, when the concentration of HA increased from 6 to 10 wt%, the crosslinking density increased and the critical frequencies (the intersection of G′ and G″) of the HPC hydrogels gradually reduced from 5.08 to 0.59 rad/s, which indicated that more compact and stable networks had finally formed.

We further employed rheological tests on the HPC-2 sample to measure its thermosensitivity. It was observed that G′ was higher than G″ at the initial temperature (Figure 2B). As the temperature increased, the hydrogen bonds in the HPC-2 hydrogel cracked, and G′ decreased obviously faster than G″, finally becoming lower than G″ when the temperature rose from 10°C to over 50°C. The inner hydrogen bond-crosslinked network disassembled, and the HPC-2 transformed to a liquid-like state. When the temperature decreased, the hydrogen bonds reformed and the HPC-2 returned to a solid-like gel state again. The reversible breakage and recombination of hydrogen bonds played an important role in this reversible thermosensitivity.

The HPC hydrogels also exhibited a typical shear-thinning behaviour. As shown in Figure 2C, when the shear rate increased, the viscosity of the HPC hydrogels obviously decreased, endowing them with good injectability. Figure 2D illustrates the letters “A” and “C” written with the HPC-2 hydrogel, further verifying its flexibility. The combination of thermosensitivity and injectability of the HPC hydrogels made it easy to add and extrude them from the syringe and then solidify them. The results suggest that HPC hydrogels have great potential in the field of biomaterials and 3D printing materials (Liu et al., 2020).

### Mechanical Properties of HA/PVP Complex Hydrogels

**FIGURE 3:**
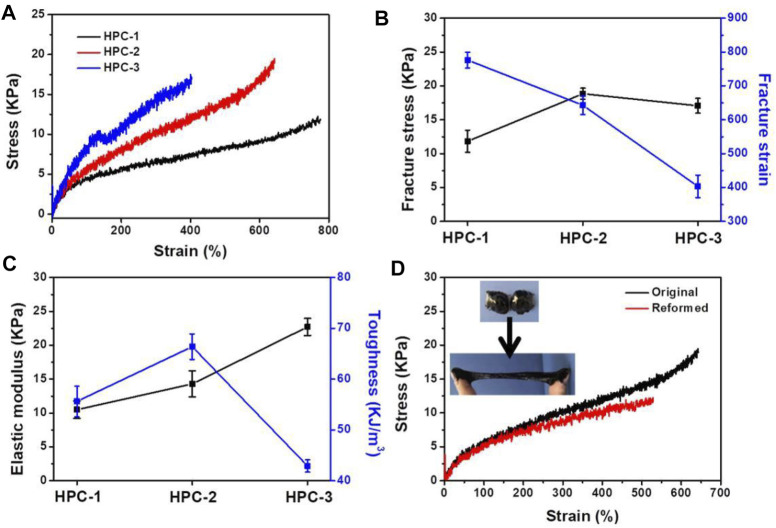
**(A)** Tensile tests of the HPC hydrogels. **(B)** Fracture stress and strain. **(C)** Elastic modulus and toughness of the HPC hydrogels. **(D)** Tensile stress-strain curves of the integrated HPC-2 sample and the reformed one.

Typical stress-strain curves of the HPC hydrogels were used to measure their mechanical properties (Figures 3A–C). We fixed the concentration of PVP at 15 wt% to test the effect of crosslinking agents of HA on the mechanical properties of the obtained HPC hydrogels. The results showed that the mechanical performance of the HPC samples was highly dependent on the HA content in the hydrogel. When the concentration of HA was 6 wt%, the fracture stress and strain of HPC-1 were 11.8 KPa and 776%, respectively (Figures 3A,B). An increased content of HA could effectively improve the crosslinking density of the hydrogel: as the concentration of HA increased to 8 wt%, the fracture stress of HPC-2 increased to 18.9 KPa, while the fracture strain decreased to 643%. In addition, the toughness of the hydrogel increased from 55.6 to 66.4 kJ/m^3^ (Figure 3C). As the concentration of HA further increased to 10 wt%, both the strength and flexibility of the HPC-3 hydrogel showed an apparent decrement, due to excessive crosslinking. The fracture stress of HPC-3 was 17.0 KPa, while the fracture strain and toughness of HPC-3 decreased to 403% and 42.9 kJ/m^3^, respectively. The elastic modulus of the HPC hydrogels increased from 10.5 to 20.7 KPa when the concentration of HA increased from 6 to 10 wt%. This might mainly be due to the over-high crosslinking density causing the mobility of the polymer chains to decrease, leading to a more rigid polymeric network and heterogeneous structure, which finally reduced the toughness (Cao et al., 2020). In general, the HPC hydrogels showed good tensile properties.

Hydrogels possessing a self-healing ability can maintain their performance and play a unique role when there is external damage, and these hydrogels are in high demand in various areas, including tissue engineering and flexible electronics (Tian et al., 2020). The dynamic and reversible crosslinking of the hydrogen bonds between HA and PVP endowed the HPC hydrogel with self-healing properties. As shown in Figure 3D, the tensile tests were applied to the original and reformed HPC-2 hydrogels to quantitatively explore the healing effect. The cracked HPC-2 hydrogel was placed at 20°C for 10 min, and the fracture stress was tested to be 11.7 KPa, which was equal to 62% of its original value. The fracture strain and toughness of the reformed HPC-2 hydrogel recovered around 80% to its original level, and the flexibility of the hydrogel was also well recovered. The insets in Figure 3D vividly show the self-healing performance of the HPC hydrogel. The two separated pieces of HPC-2 hydrogel could gradually rebuild to a single united piece within 10 min at room temperature, and the reformed HPC-2 hydrogel was easily stretched to a very large strain. The results indicate that the HPC hydrogel showed a good self-healing ability. The self-healing ability of HPC-2 hydrogel was further evaluated by alternate strains sweep of rheometer (Supplementary Figure S5). When a large strain (300%) was applied to HPC-2, the G’ dramatically decreased and was less than G”. When the strain recovered to 10%, the HPC-2 hydrogel showed rapid recovery with little hysteresis and the self-healing behavior was repeatable during the cyclic test.

### Adhesion Property of HA/PVP Complex Hydrogels

**FIGURE 4:**
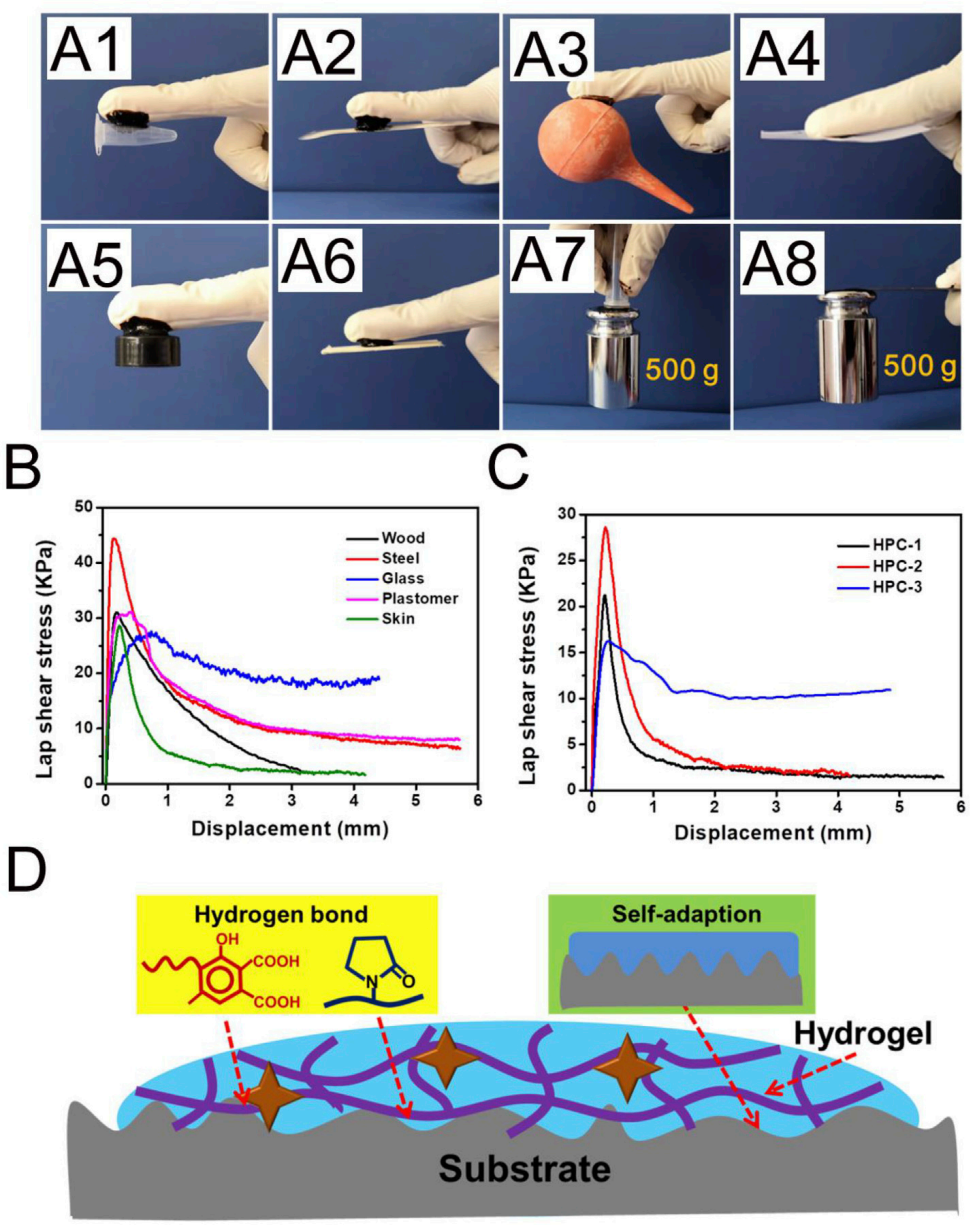
**(A)** The HPC-2 hydrogel was used as glue to tightly adhere to plastomer, steel, rubber, paper and wood substrates, and put up the weight. **(B)** Adhesion mechanical curves of HPC-2 to various substrates. **(C)** Adhesion mechanical curves of HPC hydrogels to porcine skin. **(D)** The possible adhesion mechanism of the HPC hydrogels.

Adhesion is essential for the clinical-grade employment of hydrogels. As an example, hydrogels with adhesive ability could tightly adhere to organs and tissues, which has obvious advantages in biomedical materials. As shown in Figure 4A, the HPC-2 hydrogel could act as a binder, bonding different materials, including plastomers, steel, rubber, paper and wood. PE and stainless steel sheets bonded with HPC-2 hydrogel could withstand a large load of 500 g (Figure 4A). The lap shear strengths to several typical substrates were tested in order to quantitatively evaluate the adhesion property of the HPC-2 hydrogels. Typical force-extension curves are shown in Figure 4B and indicate that the adhesion strength rapidly reaches a peak and then gradually declines in the subsequent tensile process. The adhesion strength of the HPC hydrogels to wood, steel, glass, plastomer and porcine skin varied from 27.3 to 44.3 KPa, with the best adhesion strength being to steel. The adhesive strengths of different HPC hydrogels to porcine skin were further tested to investigate how the crosslinking agents affected the adhesion property of the hydrogels (Figure 4C). When the concentration of HA increased from 6 to 8 wt%, there was an increase of adhesion strength of around 30%, from 21.4 to 28.3 KPa. As the HA content further increased, the adhesion strength of HPC-3 decreased to 16.3 KPa, indicating that the excess amount of HA hinders the adhesion properties of the hydrogel. Thus, HPC-2 was chosen for subsequent experiments. The good skin adhesion property make the HPC hydrogels suitable for use as tissue engineering materials.

According to the above experimental results, the possible adhesion mechanism of the HPC hydrogels is shown in Figure 4D. Firstly, the components in the HPC hydrogels contain a variety of functional groups, such as carboxyl, hydroxyl and amide groups. The good adhesion property of the HPC hydrogels is derived from the hydrogen bond between the functional groups, which are the source of cohesion and interfacial interaction. The hydrogen bonds existing in the HPC hydrogels work as crosslinking domains to improve the cohesion strength, while the functional groups on the interface between the hydrogel and the substrate could form hydrogen bonds and increase the interfacial interaction (Feng et al., 2021). Secondly, the soft nature endows the HPC hydrogels with good adaption ability, making it easy to fill the irregular gaps on the surface, thus increasing the contact area and the binding capacity of the hydrogel to the substrate (Yang and Yuan 2019). However, an over-high crosslinking density might cause a reduction of both cohesion strength and adaption ability, which explains why the adhesion strength of HPC-3 decreased.

### Biocompatibility Evaluation

**FIGURE 5:**
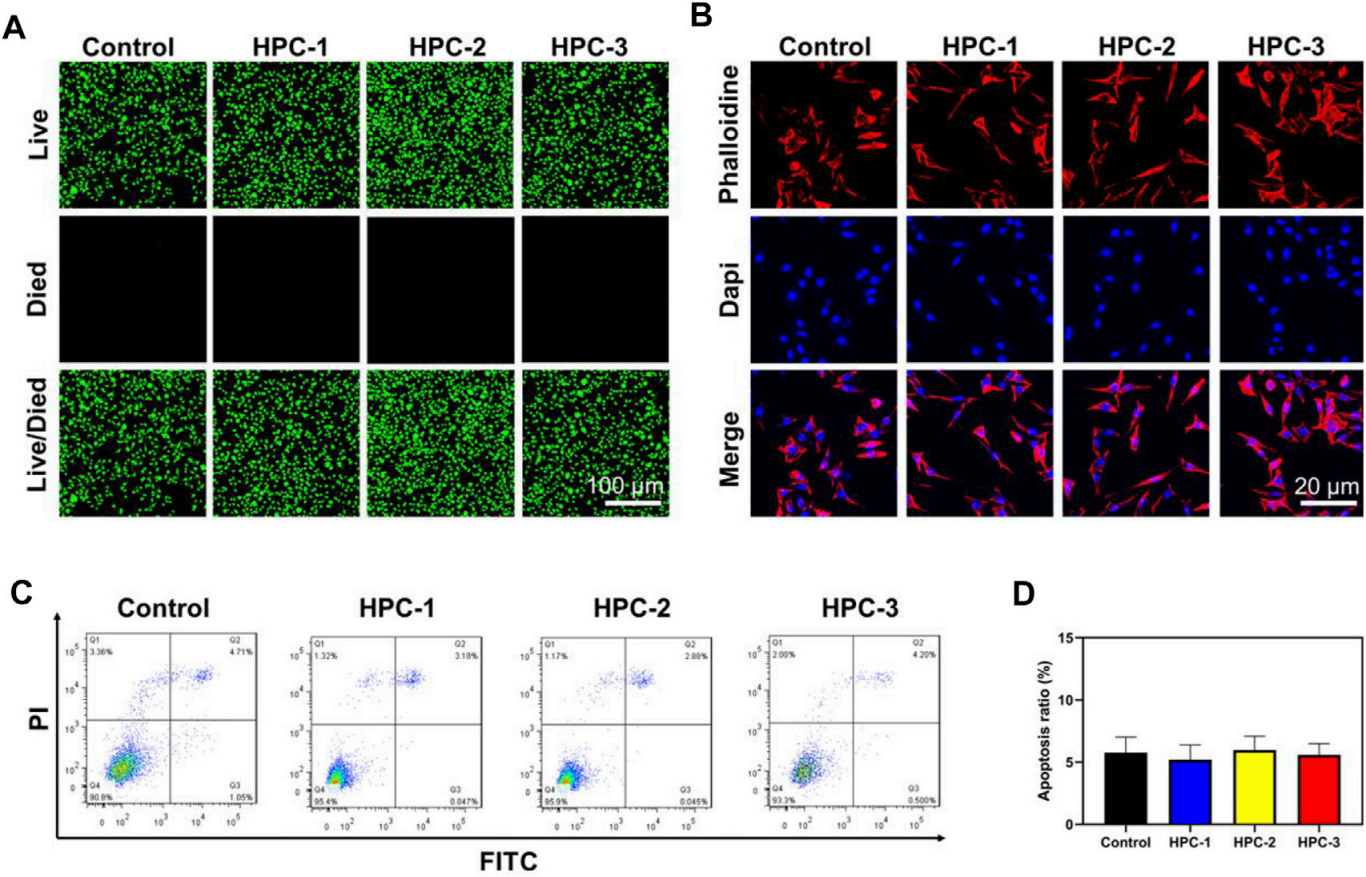
The HPC hydrogels supported the adhesion of L929 cells. **(A)** Calcein-AM/PI double staining of L929 cells treated with HPC-1, -2 and -3 hydrogels. **(B)** IF images of F-actin. Blue signal: DAPI; red signal: F-actin. **(C)** Flow cytometry (FCM) analysis and **(D)** the corresponding apoptotic rate statistical result of the HPC hydrogels.

**FIGURE 6:**
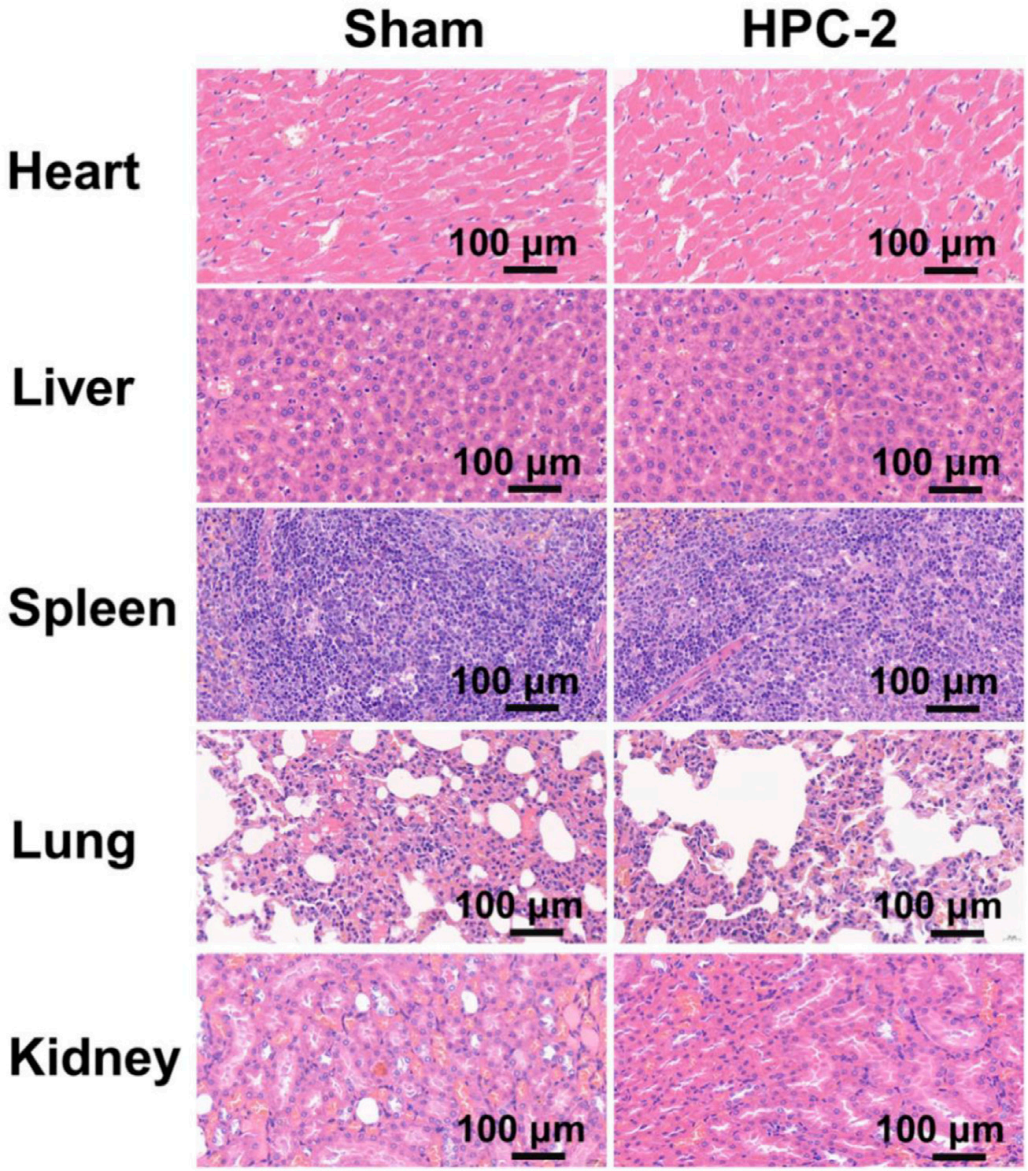
Images of haematoxylin-eosin staining of the important organs of rats treated with HPC-2 hydrogel for 14 days.

Biocompatibility is highly valued when hydrogels are used in biomedical areas. Figure 5A shows the Live/Died staining results, in which the green spots represent healthy cells, while the red spots represent apoptotic cells. In the images, a great number of green nuclei could be observed, while red nuclei were rarely observed, indicating that the HPC hydrogels showed very low cytotoxicity. To investigate the effect of HPC hydrogels on the attachment of cells, L929 cells were observed on HPC hydrogels using a confocal microscope. As shown in Figure 5B, L929 cells attached on the HPC hydrogel well, indicating that the HPC hydrogels had good cytocompatibility. We then characterized the cytocompatibility of the HPC hydrogels through flow cytometry (FCM) tests. Figures 5C,D show the results of the cells in the control groups (PBS solutions) and those cultured with HPC hydrogels, respectively. The apoptosis rates of the hydrogel-treated group were fair to the negative control, indicating that there were no additional side effects when the hydrogel samples were introduced. We further explored the biocompatibility of the hydrogels by implanting the HPC-2 hydrogel into rats and observed the HE staining results of heart, liver, spleen, lung and kidney tissues of the rats (Figure 6). Apoptosis and necrosis were barely observed in the images, and the HPC hydrogel showed good biocompatibility (Wang et al., 2022).

**FIGURE 7:**
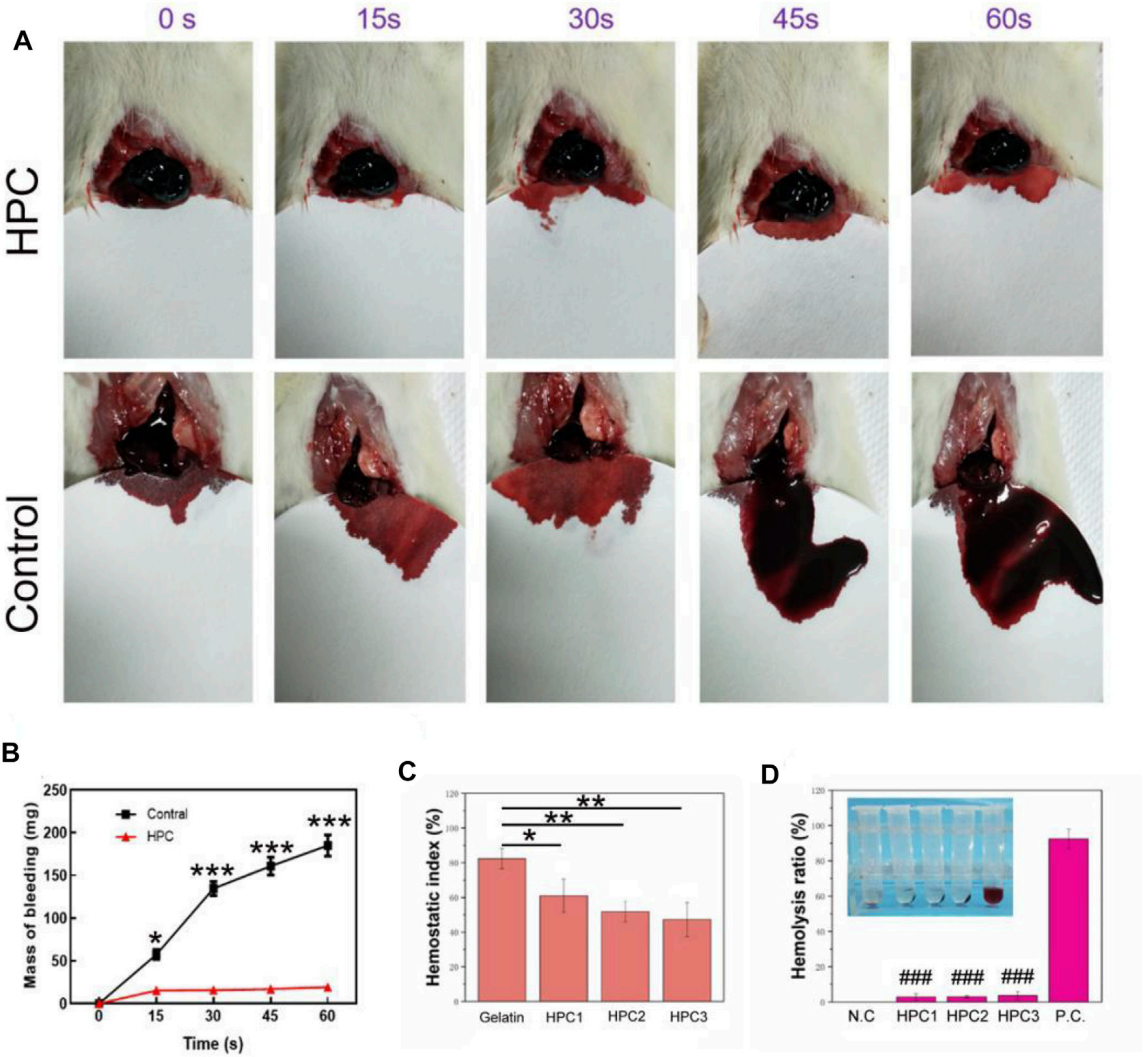
Haemostatic performance of HPC hydrogels. **(A)** Haemostatic capability of the HPC-2 hydrogel. **(B)** Mass of bleeding with or without the treatment of HPC-2 hydrogel. **(C)** Haemostatic index of control and HPC samples. **(D)** Haemolysis ratio of HPC samples (**p < 0.05, **p < 0.01, ***p < 0.001; ^###^ *p* < 0.001vs P.C. group).

The outstanding adhesive performance and good biocompatibility of the HPC hydrogels make them a promising candidate as haemostatic materials. The rat liver bleeding model was chosen as the *in vivo* animal model to investigate the haemostatic ability of the HPC hydrogels (Figure 7A). When the rat liver blood, the HPC hydrogels interacted with the damaged liver tissues, allowing the hydrogels to adhere to the haemorrhaging sites of the liver. The hydrogel could also work as a protective layer to stop the bleeding. The liver bleeding mass of the control group was 57.3, 134.7, 161 and 185 mg at 15, 30, 45 and 60 s, while the average bleeding mass with the HPC hydrogel treatment was 15.3, 15.7, 17 and 19.3 mg at 15, 30, 45 and 60 s (Figure 7B), indicating that the bleeding mass of the hydrogel-treated group decreased significantly. No excess bleeding was found at the site of injury after 1 minute of hydrogel treatment. The HPC hydrogel showed better haemostatic efficacy compared with the polyethylene glycol (PEG)-based hydrogel (control). The good haemostatic ability might be attributed to the high conglutination effect between the HPC hydrogel and the liver tissue. The haemostatic ability of the HPC hydrogel could also be monitored using the haemostatic index; a lower haemostatic index normally means a higher haemostatic ability (Figure 7C). The three HPC samples illustrated better haemostatic ability than gelatin. All in all, comparing our results to previous work, the HPC hydrogel also possessed an excellent haemostatic index as a local haemostatic agent (Li S. et al., 2020; Beaman et al., 2021).

Figure 7D shows the results of the haemolysis ratio test, which is significant for biomaterials to be applied in haemostatic areas. A haemolysis rate <5% is generally deemed to be appropriate for application in clinical haemostatic material, while a higher haemolysis ratio (>5%) suggests that the biomaterial could lead to the fracture of red blood cells. The haemolysis ratio of HPC-1, -2 and -3 were 2.8, 3 and 3.7%, respectively, demonstrating that the HPC hydrogels are nonhaemolytic materials and suitable for *in vivo* use.

## Conclusion

In this work, a typical hydrogel with injectability, good flexibility, self-healing ability, adhesion property and biocompatibility was prepared. The HA molecules worked as a crosslinking agent to form hydrogen bonds with PVP and induced the solution to gelation. The whole synthetic process was direct and lasted less than 10 s. The mechanical properties of the HPC samples were regulated by changing the HA content. With a gradual increase in HA content, the HPC hydrogels showed improved mechanical properties at the beginning, and the maximum value of the fracture stress reached 18.9 KPa. Excess HA led to a brittle network and a decreased fracture stress due to the over-high crosslinking density. The dynamic and reversible crosslinking of the hydrogen bonds between HA and PVP also endowed the hydrogel with a self-healing ability and injectability. When encountering large deformation, the internal structure of the hydrogel was damaged and could rapidly recover to its original state. The HPC hydrogels also showed good adhesion, which was attributed to the abundant hydrogen bonding and adaptation to the interfaces, and good biocompatibility. Taking advantage of all the above characteristics, the HPC hydrogels were used as haemostatic agents to treat damaged tissue, where the quantity of bleeding obviously decreased, from 185 to 19.3 mg, when injured liver was treated with HPC-2 hydrogel, showing an efficient haemostatic effect. Overall, the HPC hydrogel would be a good alternative to the current haemostatic dressings and could be widely applied in the biomaterials field.

## Data Availability

The original contributions presented in the study are included in the article/Supplementary Material, further inquiries can be directed to the corresponding authors.
