# Supplementary material for: A Hydrogen Bonds-Crosslinked Hydrogels With Self-Healing and Adhesive Properties for Hemostatic
Source: Front Bioeng Biotechnol. 2022 Apr 14;10:855013. doi: 10.3389/fbioe.2022.855013 (PMC9046721; doi:10.3389/fbioe.2022.855013)
Supplement: Supplementary file 2 [file DataSheet1.PDF]

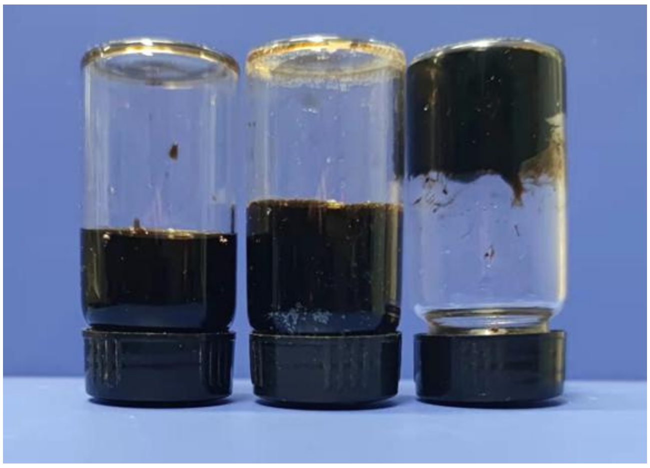

Figure S1. The compounds of HA and PVP (From left to right, the molecular weights of PVP were 8×10^3, 2.4×10^4, and 1.3×10^6 g/mol, respectively).

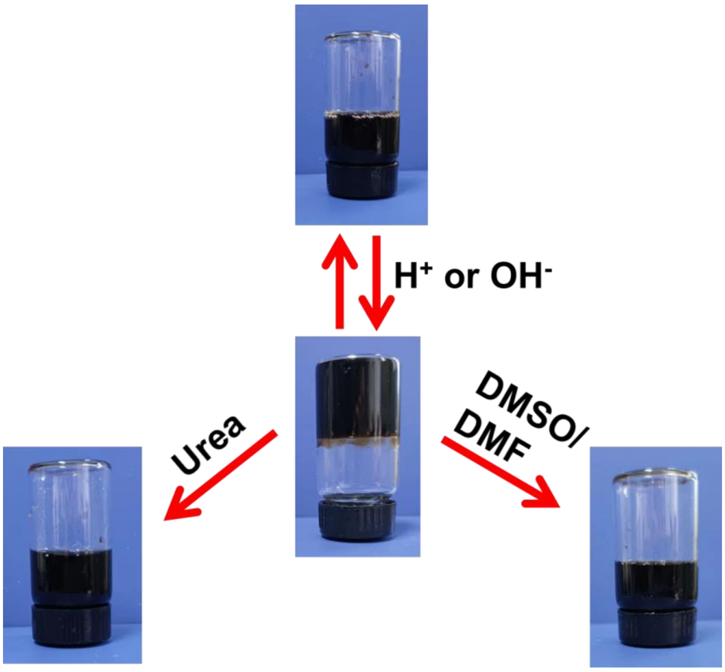

Figure S2. The responsivity of HPC hydrogel to various solvents.

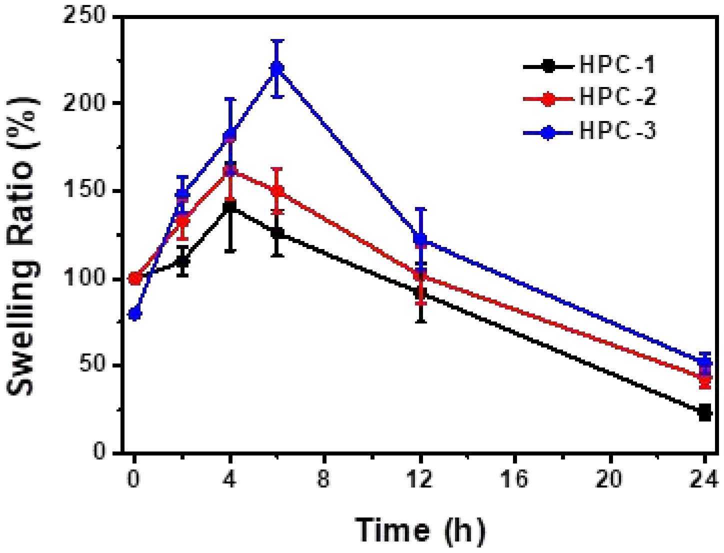

Figure S3. The swelling ratios of HPC hydrogels in water.

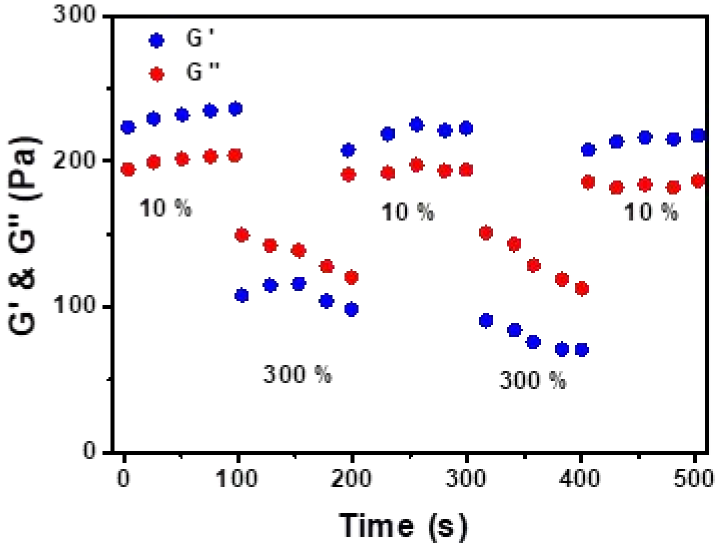

Figure S4. Alternate strains sweep to HPC-2.
